# Animal Models and Their Contribution to Our Understanding of the Relationship Between Environments, Epigenetic Modifications, and Behavior

**DOI:** 10.3390/genes10010047

**Published:** 2019-01-15

**Authors:** Natalia Ledo Husby Phillips, Tania L. Roth

**Affiliations:** Department of Psychological and Brain Sciences, University of Delaware, 108 Wolf Hall, Newark, DE 19716, USA; nphillips@psych.udel.edu

**Keywords:** animal models, environments, epigenetics, behavior, disease

## Abstract

The use of non-human animals in research is a longstanding practice to help us understand and improve human biology and health. Animal models allow researchers, for example, to carefully manipulate environmental factors in order to understand how they contribute to development, behavior, and health. In the field of behavioral epigenetics such approaches have contributed novel findings of how the environment physically interacts with our genes, leading to changes in behavior and health. This review highlights some of this research, focused on prenatal immune challenges, environmental toxicants, diet, and early-life stress. In conjunction, we also discuss why animal models were integral to these discoveries and the translational relevance of these discoveries.

## 1. Introduction

**Figure 1:**
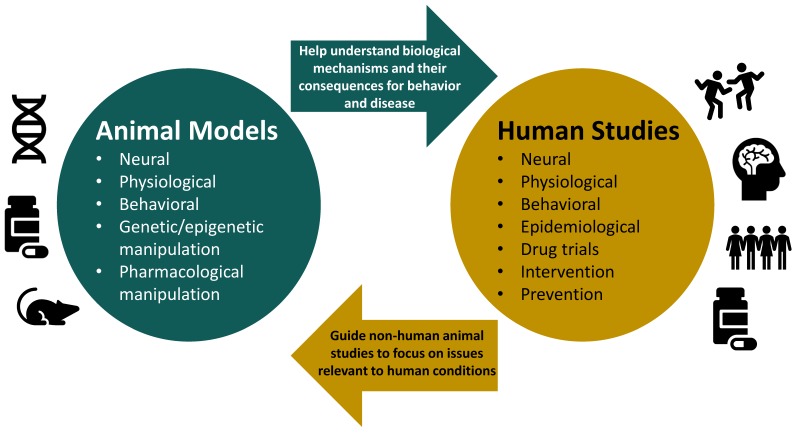
The relationship between animal models and human studies is reciprocal: both provide important insight into biology and behavior, guide the direction of one another’s research, and complement each other’s findings. With non-human animal models, however, we can utilize genetic, epigenetic, and pharmacological manipulations to help elucidate mechanisms and establish causality between environmental factors and behavior, health, and disease outcomes.

For more than 2000 years, humans have used animal models (e.g., dogs, chicks, pigs, rats, monkeys) to understand our own biology, behavior, and health [1]. Animal models are incredibly important because they allow researchers to elucidate complex biological mechanisms that underlie development, behavior, and health (Figure 1). This is something that cannot be done in human research because elucidating such requires carefully controlled experimental manipulations, such as genetic knock-out/knock-ins, hormone alterations, administration of pharmacological agents, or exposure to controlled environmental toxicants or viruses, and then the collection of brain tissue for biochemical analyses. Furthermore, animal models help control environmental variability that exists in epidemiological and clinical research [2] and afford the opportunity of carefully controlled behavioral observations and measurements. In research involving human participants, it is difficult and sometimes impossible to disentangle the contribution of different factors in an individual’s life to physiological, behavioral, and health outcomes. On the other hand, animal models allow researchers exquisite control of all the factors that the animal is exposed to and to test the effects of factors individually or together [2]. Parsing apart the downstream consequences of a specific factor or a group of factors as well as correlational versus causative impact is critical to continue the forward momentum in developing precision medicine and treatments [3].

Numerous studies have illustrated the importance of both genetic and environmental factors on biology, behavior, and health, and early evidence emerged suggesting that genes and the environment somehow interact to produce various behavioral and disorder or disease phenomena [4,5]. We now understand this interaction to occur via epigenetics, a process by which environmental experiences induce changes in gene activity without modifying the underlying DNA sequence [6,7]. Epigenetic alterations lead to changes in biological functions within the organism, and subsequently, changes in behavior or health [8,9]. These modifications can occur at any point in an organism’s life, although early development appears more vulnerable, and can even be heritable [6,10].

This review highlights animal model discoveries in the realms of prenatal immune challenges, environmental toxicants, diet, and early-life stress that have significantly advanced our understanding of the relationship been environments, epigenetic modifications, behavior and health. The translational relevance of these discoveries is addressed, as well as how animal work is informative of what researchers should study in humans. An additional theme is how human work has guided animal research, allowing scientists to delve further into the causal factors of environmental experiences and biological characteristics that contribute to abnormal brain development and disorder or disease.

## 2. Prenatal Immune Challenges

In the last two decades, studies provide evidence that infections during pregnancy, or maternal immune activation (MIA), lead to epigenetic and behavioral changes in offspring, with some of these same changes observed in individuals with schizophrenia and Autism Spectrum Disorder (ASD). For example, epidemiological research suggests that viral or bacterial infections during early or mid-pregnancy increase risk for neurodevelopmental disorders such as ASD and schizophrenia by 2- to 7-fold, two disorders in which genetics is clearly involved [11,12,13,14,15,16,17]. Experimental models of infection have been necessary to elucidate the causal and mechanistic links between early MIA and altered neurodevelopmental and health outcomes. Such studies indeed reveal behavioral and cognitive abnormalities in adult mice and rats with prenatal exposure to for example, bacterial endotoxin lipopolysaccharide (LPS), human influenza virus, or a viral mimic called polyriboinosinic-polyribocytidilic acid (Poly(I:C)) [15,16,18,19]. Depending on the time of exposure, rats demonstrate different cognitive and behavioral deficits, with early and mid-pregnancy exposure being the most debilitating on these outcomes [15].

While schizophrenia and ASD are disorders with distinct characteristics, they do show similarities in social and cognitive dysfunction. For example, individuals with ASD or schizophrenia both have difficulties with social cues and social interactions. They also show deficits in prepulse inhibition (PPI) and latent inhibition (LI) [15]. Both LI and PPI are used as measures of whether an organism can filter out irrelevant stimuli. Deficits in PPI and LI indicate disrupted information processing. PPI refers to a decreased response to a startle stimulus, when the startle stimulus is preceded by a moderate neutral stimulus [20]. LI refers to a decreased ability to learn the relevance of a stimulus that is paired with an aversive or positive condition through classic conditioning if there has been a previous exposure with the stimulus in a neutral context [21,22]. Rodents also show LI and PPI, which makes them a suitable model for testing whether and how deficits in PPI and LI reflect environmentally-driven changes in the brain. Rodents are also very social animals, enabling scientists to use them as models of social behavior. Furthermore, both schizophrenia and ASD share many of the same genes that have been identified as at-risk for the development of these disorders [23,24,25], and importantly, humans share almost all protein coding DNA with rodents, especially rats [26].

Since both human and animal model studies indicate that MIA can lead to substantial alterations in fetal brain development, often leading to behavioral dysfunction, much recent attention has focused on understanding the role of cytokines in neurodevelopment [18,27,28,29,30]. Cytokines are substances secreted by cells of the immune system and are increased in production in response to infection [16]. Rats exposed to the cytokine interleukin-6 (IL-6) in utero display deficits in PPI, LI, and social interaction [18,19], all deficits commonly observed in rodent models of ASD or schizophrenia. The mechanisms by which IL-6 does this are still largely unknown, but this cytokine crosses the placenta with more ease than others, suggesting that it could be more detrimental to neurodevelopment than other cytokines [31]. Furthermore, the permeability of the placenta to IL-6 varies depending on the time of pregnancy; fetuses of rat dams who were injected in mid-pregnancy (E11-13) had higher levels of IL-6 compared to offspring of dams who were injected in late-pregnancy (E17-19) [27].

Emerging evidence points towards epigenetic modifications [16,19,28] as a route through which IL-6 influences development and behavior. Hodge et al. [28] provided seminal in vitro evidence that IL-6 has consequences for DNA methylation. DNA methylation is the attachment of a methyl group to a cytosine on the DNA strand and is made possible by enzymes called DNA methyltransferases, or DNMTs [reviewed in 6]. DNA methylation is typically associated with gene silencing through the recruitment of co-repressor proteins and the blocking of transcription factors [6]. In vitro, IL-6 enhances translocation of enzyme DNMT1 to the nucleus [28], which could produce aberrant DNA methylation. But does IL-6 have this same effect in vivo and what consequences does this effect have for development? Wu and colleagues [19] investigated pathways in which IL-6 might disrupt fetal neurodevelopment by creating a knockout mouse line that lacked IL-6 receptors in the placenta (pIL-6R). Pregnant dams were exposed to Poly(I:C) during mid-pregnancy (E12.5) and expression of STAT3 in the fetal brain was measured three hours post-injection. STAT3 is a protein that gets phosphorylated by Janus kinases (JAK) in response to cytokines, acting as a transcription factor to mediate the expression of many genes. Wu et al. [19] found that STAT3 expression was increased in the fetal brain of wild-type mice. Furthermore, increased STAT3 activity led to increases in *Myc* and *Stat3* expression. Increased *Myc* expression is observed in individuals with ASD [32], and thus animal research indicates it would be useful to explore whether individuals with ASD that have increased *Myc* expression also have mothers who went through an infection during pregnancy. Importantly, pIL-6R KO mice did not show an increase in IL-6 or STAT3 in the fetal brain, a finding that indicates that IL-6 receptors in the placenta are necessary for STAT3 activation and subsequent changes in gene expression in the fetal brain. MIA wild-type mice also had decreased sociality and increased anxiety-like behavior as measured by the three-chamber social and marble burying tests [19].

Viral mimetic Poly(I:C) exposure in mid- (E9) and late-pregnancy (E17) alters global methylation within the prefrontal cortex (PFC) [30]. Mice exposed at E9 had 2365 differentially methylated CpG sites compared to controls in adulthood. E17-exposed mice had 3361 differentially methylated sites. Methylation alterations in both E9 and E17 were enriched for genes involved in brain development, synaptic plasticity, and neuronal differentiation. Furthermore, differentially methylated genes also had corresponding altered mRNA levels, indicating that prenatal infection had a functional consequence on gene expression. Labouesse et al. [33] also utilized a Poly(I:C) mouse model of prenatal infection to examine whether MIA affects DNA methylation, hydroxymethylation, γ-Aminobutyric acid (GABA) function and behavior. Hydroxymethylation of DNA occurs when a methylated cytosine is oxidized by ten-eleven translocation (TET) proteins [34]. The role of hydroxymethylation in gene regulation can vary from a transient step in the process of demethylation, to playing a stable role in epigenetic regulation [35,36,37]. Its role likely varies depending on developmental age, tissue type, and genomic region [34]. MIA increases methylation and hydroxymethylation of cytosines within the PFC, specifically at *GAD1* and *GAD2* promoters [33], 2015). *GAD1* and *GAD2* are genes that code the GABA-synthesizing enzyme, glutamic acid decarboxylase (GAD67 and GAD65). MIA also increases binding of methyl CpG-binding protein 2 (MeCP2) at *GAD1* and *GAD2* promoters and decreases GAD67 and GAD65 mRNA expression.

Finally, we are learning that MIA has long-lasting behavioral and epigenetic consequences that span generations [16]. Using the Poly(I:C) mouse model of MIA, Weber-Stadlbauer and colleagues [16] demonstrate decreased sociability, increased cued fear expression, and behavioral despair in third-generation mice (paternally-derived). Differentially-expressed genes involved in both glutamatergic and dopaminergic-signaling pathways may be responsible for the phenotypes. In sum, epidemiological studies informed the field of neuroscience that infection during pregnancy was likely associated with disorders such as schizophrenia and ASD. This indicated to scientists the necessity to develop rodent models of these infections to determine whether infection was causally contributing to the development of disorders. Another observation made possible because of rodent models is that of the multigenerational consequences of maternal infection. Because of the discoveries it may be possible to develop pharmaceutical agents that block the effects of infection on the fetal brain, potentially preventing individuals from developing either schizophrenia or ASD.

## 3. Environmental Toxicants

With the increase in chemical pollutants in our society, the widespread use of plastics in everyday household items, and the use of pesticides in both mass production and local agriculture, we are increasingly exposed to chemicals that modify our epigenome [38]. Bisphenol chemicals (BPA, BPAF, and BPS) and heavy metals such as lead, and pesticides (such as vinclozolin) are known to impact epigenetic and behavioral states [39,40,41]. The prenatal period of development is extremely sensitive to such external factors, as it is a period of dramatic growth and differentiation of brain cells [41,42]. Particularly, neurogenesis, neural migration and maturation are processes dependent on epigenetic control, making offspring highly susceptible to the influences of bisphenol chemicals, lead, and vinclozolin [42]. In fact, epidemiological research links these toxicants to certain epigenetic and behavioral outcomes [41,43].

BPA, BPAF, and BPS are classified as endocrine-disrupting chemicals (EDCs), as they bind to estrogen receptors ERα and ERβ, activating signaling cascades that lead to changes in gene expression [44,45,46,47]. BPAF and BPS were originally created as substitutes for BPA but have been found to have stronger effects than BPA [46,47]. In addition, both are often used in everyday products. Studies in humans indicate that BPA is detectable in urine, fetal placental tissue, cord blood, and fetal tissue [48,49,50]. To understand how bisphenol chemicals can lead to changes in the brain, Li and colleagues [45] conducted an in vitro study to test the effect of BPA and BPAF on three different cell lines. Some of their observations include BPA and BPAF acting as either agonists or antagonists to ERs in a dose-dependent manner, these agents activating different genes, and these agents having effects dependent on cell line, which together are all indicative that these chemicals can disrupt endocrine signaling by way of many different mechanisms. BPS binds selectively to ERα, and recruits more co-repressors of transcription than either BPA or BPAF, suggesting that BPS could have a more profound influence on gene expression, and possibly more severe effects to those who are exposed to it [46]. Evidence from an in vitro study shows apoptotic effects of BPAF on hippocampal neurons [51].

While in vitro studies are a great first step in understanding the consequences of toxicant exposure in a precisely controlled environment, it is important to explore their consequences under more realistic conditions where multiple cells are constantly communicating with one another and the organism is regularly interacting with its environment (i.e., in vivo). In vivo activation of ERα and ERβ leads to a host of changes in gene expression throughout the brain. Cao et al. [52] examined the effects of BPA on gene expression of *ERα*, *ERβ*, and *Kiss1* within the hypothalamus. Neonatal rats (PN1–PN10) exposed to BPA have altered expression of all three genes. During the neonatal period, the hypothalamus undergoes steroid-directed sexual differentiation. Thus, endocrine disruption at this time could induce permanent, lifelong changes in the brain and body. The rat neonatal period is the equivalent of the third trimester in pregnancy, so future studies should examine whether high maternal exposure to BPA in late pregnancy is related to changes in the same genes of the fetus. One way to measure this is by measuring gene expression in cord blood [43].

Much of the bisphenol chemical literature focuses on whether these substances activate ERs, and whether this leads to changes in gene expression. Studies are beginning to elucidate the influence of these substances on epigenetic mechanisms such as DNA methylation as driving factors of the change in gene expression and behavioral and health outcomes. For example, Dolinoy et al. [8] investigated how maternal exposure to BPA affects DNA methylation and phenotypes of Agouti mice offspring. In utero BPA exposure yields a yellow-coated phenotype in offspring. A yellow coat phenotype occurs when the mouse is heterozygous for the agouti yellow allele. Mice with this phenotype are more prone to health problems such as obesity and diabetes [8]. BPA exposure also decreases DNA methylation in the *Agouti* gene. Hypomethylation and the yellow coat phenotype can be prevented by administering methyl donor supplements (e.g., folic acid and genistein) in the diet of BPA-exposed dams. Kundakovic et al. [43] also show consequences of BPA exposure on DNA methylation and do so in both rodents and humans. Male mouse offspring were most vulnerable to the effects of BPA exposure in utero, showing hypermethylation of hippocampal *Bdnf* (brain-derived neurotrophic factor) DNA, concomitant reduced gene expression, and behavioral deficits in a novel object recognition test (NOR). Lastly, cord blood samples from human mothers with high exposure to BPA during pregnancy likewise show hypermethylation of *Bdnf* DNA methylation, providing direct translational relevance of the rodent data. Epidemiological research on BPA reveals sex-specific effects: some studies show more severe behavioral deficits such as hyperactivity and anxiety in female children exposed to BPA in utero, whereas others showed that BPA was more behaviorally disruptive to males [41]. It is difficult, however, to tease apart the effect of bisphenol chemicals from other factors that could contribute to the behavior of these children.

Lead exposure is severely disruptive in early development (but can also have deleterious effects when encountered throughout the lifespan). Postnatal exposure to lead is associated with increased risk for neurodevelopmental disorders and disruptions in cognition [41]. Lead can also cause encephalopathy at high levels (70 μg/dL, measured in blood). In fact, even at 56 μg/dL, lead leads to severe brain damage in infants [53]. In rodents, lead downregulates synaptic genes, lead to alterations in synapses, and increases amyloid beta 40 (aβ40) [41]. Increased aβ40 is especially concerning because this fibril makes up the plaques that form in the brains of Alzheimer’s patients. Rodents exposed to lead have alterations in DNA methylation regulators (for example DNTM1 and DNTM3a), hypermethylation of numerous genes within the hippocampus, and deficits in memory formation [25]. Interestingly, similar alterations in DNA methylation regulators following lead exposure have been observed in *Macaca fascicularis* [30]. Future studies could examine whether these same changes also occur in humans exposed to lead. In human embryonic stem cells, lead disrupts neuronal differentiation and induces global hypermethylation [38].

Pesticides are chemicals used to protect crops from pests, weeds, and diseases, particularly vector-borne diseases such as dengue fever or malaria [54]. Pesticides are also used in urban green areas, sports fields, pet shampoo and building materials [54]. Thus, the level of exposure to pesticides can vary considerably depending on a person’s environment and lifestyle. Human studies suggest that exposure to pesticides can disrupt cognition and increase the risk for cancer, asthma, diabetes, Parkinson’s disease, and leukemia [55]. Rodent studies help us realize that these health problems are attributable to pesticide exposure and can even be passed through generations [39,56,57,58].

Vinclozolin is an endocrine disruptor and a common pesticide used in crops. Illustrating its damaging consequences for the epigenome and somatic health, rat pups exposed in utero from E8-14 display symptoms of kidney disease, prostate disease, immune system abnormalities, testis and reproductive health abnormalities, and cancer [56,57]. What is remarkable is that some of these health consequences are even present in fourth-generation offspring, offspring certainly never directly exposed to the pesticide. The pesticide and bug repellent mixture (permethrin and DEET) likewise has similar effects, including differential methylation patterns in the sperm of exposed males [58].

In sum, the dangers of gestational and early postnatal exposure to environmental toxicants is clear, and animal models have helped us realize their multigenerational consequences. These discoveries are informative for policy research aimed at reducing or preventing toxicant exposure. They are also suggestive of research avenues for strategies that could counteract the damaging effects of toxicant exposure.

## 4. Diet

Dietary nutrients that we ingest provide precursors and methyl groups for the process of DNA methylation. The primary source of methyl groups for DNA methylation is s-adenosylmethionine (SAM), which is catalyzed when nutrients and vitamins such as folate, vitamin B, and choline are present in the diet [59,60]. Nutrients such as folate can alter DNA methylation in a gene and tissue-specific manner at different life stages [7,59,61,62]. In humans, folate, or folic acid, is the most studied regarding prenatal development [59,63], with folic acid supplementation prior to conception and during pregnancy a well-validated recommendation to significantly reduce the risk of neural tube defects [63]. Animal models have been instrumental in showing the necessity of folate for nucleic acid synthesis and methyl group bioavailability in preventing the prevalence of neural tube defects [7,8,64,65].

Intrauterine exposure to nutrient restriction likewise has epigenetic and phenotypic consequences, most famously evidenced by the Dutch Winter Famine from 1944–1945 [12,66,67,68]. Animal experiments provide empirical support for epigenetic mechanisms as the link between maternal diet and phenotypic outcome [8,65]. For example, scientists showed that a methyl-rich diet during pregnancy influences coat color and feeding behavior in offspring [65]. Methyl donor-supplemented diets outside of sensitive periods of development also influence DNA methylation with consequences for behavior [64]. Methyl donor supplementation for 18 weeks in adult rats can mitigate the effects of early-life stress on hippocampal DNA methylation, cholesterol levels, and forced swim behavior. Folate and methyl-deficient diets in adulthood are also known to alter brain methylation [7,60].

Animal research also helps us see that paternal diet prior to conception has an influence on offspring development and behavior [69,70]. McCoy et al., [70] depleted dietary methyl-donors of anxiety-prone male rats for five weeks prior to mating. Fathers exhibited exacerbated anxiety-like and depression-like behaviors in the open field and forced swim tests, and their offspring showed similar behavioral profiles. Lambrot et al. [69] fed male mice a low folate diet and found that their sperm had differential methylation of genes associated with a variety of mental disorders such as schizophrenia, autism, as well as genes associated with development, cancer, and diabetes. Offspring of these males showed more birth defects.

Nutrition outside of sensitive periods of development likewise has physiological and pathological consequences, and animal work illustrates the ability of diets to produce changes in the epigenome. A chronic high-fat diet (HFD) leads to altered physiological responses to stress, an effect involving changes in gene regulatory responses regulating stress responsivity and inflammation [71]. An HFD can also alter methylation and gene expression within brain reward circuitry [62]. Shen et al. [72] induced obesity in mice via a diet in which 60% of calories came from fat. They found that diet-induced obesity (DIO) mice increased MeCP2 and DNMTs in adipose tissue at the leptin promoter region (a gene that helps regulate appetite), as well as hypoacetylation of histones and increased binding of histone deacetylases at the same gene region. Histones are alkaline proteins that DNA wraps around and modifications to histone tails helps regulate gene activity [73]. DIO can also alter DNA methylation of memory-associated genes, including *Sirt1* within the hippocampus [74], an effect that can be reversed by resveratrol.

In sum, nutrients and diets all influence behavioral and health states of humans, with animal work making it possible for us to see how this involves changes to the epigenome. Animal work also helps us see clearly that is important to find a healthy balance, as too much or too little of nutrients can have deleterious effects. Such evidence should make individuals wary of committing long-term to particular unbalanced diets. Based on data from animals fed a high-fat diet, it would be interesting to examine the effects of the popular keto diet on DNA methylation in human participants.

## 5. Early-Life Stress

Stress and adversity encountered during early development are major risk factors for the development of cognitive and emotional disorders [75,76,77,78]. An increasing number of studies link such developmental experiences to changes in the epigenome [79,80,81,82]. Gene systems in general that seem especially sensitive include those helping to regulate neural development, stress responsivity, and inflammation. Data gathered from various rodent models were the driving impetus to examine DNA methylation in these cited human studies (and many others we do not have room to include) as well as non-human primate studies [83]. Animals models have been vital in providing clear experimental evidence that early caregiving environments and experiences do indeed affect DNA methylation with functional consequences for behavior, as the design of human studies and the unavoidable lack of variable control leave us unable to determine whether specific prenatal or postnatal experiences are causally responsible for the observed changes in DNA methylation, behavior, and health.

In rodents, maternal input is the major environmental experience (as fathers are typically removed from the home cage after breeding), and research with animal models has shown us that the quality of maternal care influences pup outcomes via epigenetic alterations. In a seminal study in the field of behavioral epigenetics, Weaver et al. [84] showed that levels of maternal care (low licking/grooming (LG) vs. arched-back nursing (ABN) vs. high LG and ABN) alter methylation at the glucocorticoid receptor (GR) promoter in the hippocampus of offspring. The maternal influence on methylation (and histone acetylation) can persist into adulthood and is associated with altered stress responsivity, but importantly, is modifiable (achieved in their study via cross-foster care or HDAC inhibition). For example, adult rats who experienced low LG/ABN but were given an HDAC inhibitor had their GR expression and stress response normalized. Demonstrating translational relevance of these findings, McGowan et al. [85] found increased methylation of *NR3C1* gene (human equivalent of GR), decreased *NR3C1* expression, and decreased NGFI-A binding in the hippocampus of individuals with a history of child abuse. In similar fashion, mean *NR3C1* DNA methylation levels are increased in depressed individuals with a history of abuse, with methylation at specific CG sites within *NR3C1* exon 1F related to childhood emotional abuse severity [86].

Animal models often utilize either maternal separation or limit nesting resources to simulate early adversity [87,88,89,90,91]. While some maternal separation studies support evidence that early-life stress affects the epigenome [64,90,92], others report conflicting results [10,93,94]. On the other hand, using the limited nesting resources model in rodents has proven to produce more consistent and reproducible findings across labs [95]. This model has another strength: it is more naturalistic compared to maternal separation [75]. It is also relevant to humans because it simulates conditions of poverty, a very prevalent global issue [75]. Rat dams given limited nesting material have fragmented and unpredictable behaviors such as poorly-treating pups, leading to elevated corticosterone levels in the pups [96,97,98,99].

Roth et al. [91] used a limited nesting resource model to examine the effects of maternal maltreatment on the offspring’s epigenome. Their data show us that even brief exposure to mild adversity during a sensitive period of development can increase cortical DNA methylation and suppressing gene expression (of *Bdnf*). Furthermore, the experience of maltreatment alters maternal behavior (leading females to maltreat their own offspring), with some changes in DNA methylation likewise present in the next generation. Lastly, the maltreatment-induced gene changes can be mitigated with strategies that alter DNA methylation (in this study, they used a drug known to alter DNA methylation called zebularine). Keller, Doherty, and Roth [86] recently extended these findings to show zebularine administration in adulthood can also improve some of the behavioral deficits caused by maltreatment (forced swim behavior in this study). Together, work from various animal models show that environmental or pharmacological interventions are beneficial to individuals who do not receive adequate care early in life, just like you see in the clinical literature, but provide insight into the brain mechanisms of necessary targets.

Prenatal exposure to psychosocial stress also has consequences for offspring development and health [100,101,102,103] and examples from the human literature suggest that maternal prenatal stress is related to changes in offspring methylation. For example, Cao-Lei et al. [102] did a population-based study on the effects of the Quebec ice storm on experiences of hardship and distress of pregnant women, and how their experiences were associated with child outcomes. Thirteen years later, they observed that prenatal maternal hardship correlates with methylation levels in 1675 CpGs affiliated with 957 genes that are involved in immune function. Animal work is what provides the causal link between prenatal stress, epigenetic modifications, and behavior. In 2008, a seminal investigation highlighted the role of epigenetic mechanisms in the maladaptive effects of prenatal stress on offspring HPA responsivity and behavior [104]. Blaze et al., [100] found hypermethylation of *Bdnf* DNA in the medial prefrontal cortex (mPFC) of adult offspring exposed to prenatal stress (pregnant rat dams were exposed to unpredictable and variable stressors during pregnancy). Peña et al. [103] examined prenatal stress and its effects on placental 11β-hydroxysteroid dehydrogenase type 2 (HSD11B2). HSD11B2 is an enzyme that protects the fetus from maternal stress by converting cortisol and corticosterone into inactive metabolites. However, chronic stress during pregnancy can downregulate this enzyme. Peña et al., [103] found that prenatal stress increases methylation of HSD11B2 promoter DNA, with changes in methylation also present in the fetal hypothalamus. Together, these results provide experimental evidence of a mechanism whereby stressors in the womb can affect behavioral and health trajectories.

Lastly, animal work has shown the multigenerational transmission of epigenetic marks and behavior/health associated with developmental stress [85,91,105,106,107,108]. In some instances, investigators have discovered epigenetic changes in the germline as a result of developmental exposure to stress and adversity [106,109,110]. These discoveries are remarkable, demonstrating a mechanism by which the effects of such experiences are transferable across generations. They argue for better policies and practices concerning child development and health. They also help us see that early or traumatic experiences and resultant epigenetic changes should not be viewed as determinative, as there is malleability in our systems (epigenetic and brain) that can be capitalized on to improve behavior and health.

## 6. Concluding Remarks and Recommendations for Future Research

**Figure 2:**
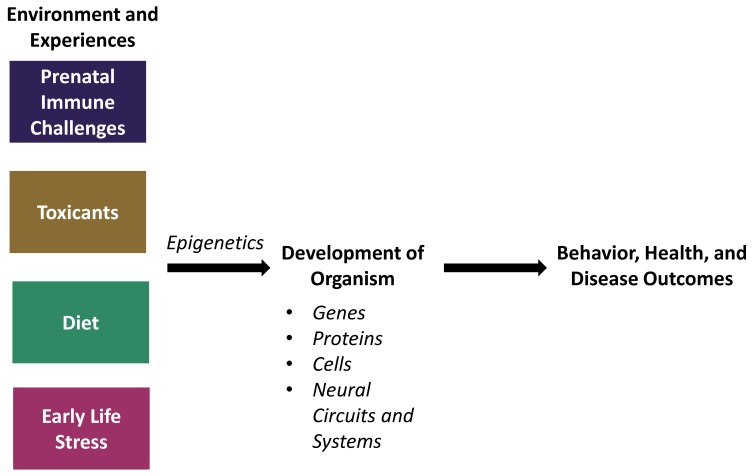
Factors in the environment in addition to experiences throughout the lifespan can influence the development of an organism via epigenetic mechanisms, thus altering subsequent behavior, health, and disease outcomes.

Without the use of animal models in science, biopsychology and behavioral neuroscience research would be largely limited to correlational studies. While correlational studies are a good starting point, they cannot establish causality. Thus, animal models are necessary to determine whether and how certain factors contribute to our biology, behavior, and health. Recent work in the field of behavioral epigenetics with various animal models has certainly started to give us a better understanding of the complex mechanisms that account for the relationship between brain development and behavioral and health outcomes. We have described evidence for how four different factors affect biology and behavior in both humans and non-human animals, and how epigenetic mechanisms mediate these outcomes (Figure 2).

However, it is rarely the case that these factors happen exclusively. For example, children who grow up in low socioeconomic status have altered immune function [111], are often exposed to more environmental toxicants [112], suffer poor nutrition [113], and are exposed to various psychosocial stressors [114]. Similarly, children who experience institutionalization are affected by a pervasive mixture of these elements [111,115,116,117]. While the animal models reviewed in previous sections have helped us understand how prenatal immune challenges, toxicant exposure, dietary factors, and early-life stress can each affect brain and behavior, future studies are certainly warranted that incorporate multiple factors to help elucidate how factors act in conjunction to influence development. Such integrative models are likely to reveal novel mechanisms with important translational relevance. As we continue to elucidate such mechanisms, we will also discover novel strategies (pharmacological and/or behavioral) to treat or even prevent certain diseases and disorders.
